# Effectiveness of the Natural Breast Self-Circulation Program on Postpartum Breast Engorgement, Mental Health, and Quality of Life Among Early Postpartum Women: A Quasi-Experimental Study

**DOI:** 10.3390/healthcare14091158

**Published:** 2026-04-25

**Authors:** Ohsuk Hwang, Miran Jung

**Affiliations:** 1 Dongtan Jeil Women’s Hospital, Hwaseong 18450, Republic of Korea; baby7mom@naver.com; 2 Department of Nursing, Baekseok University, Cheonan 31065, Republic of Korea

**Keywords:** breast engorgement, anxiety, massage, self-care, postpartum period

## Abstract

**Highlights:**

**What are the main findings?**

- Early postpartum breast care is critical for maternal and neonatal health.
- Our program (the natural breast self-circulation program) effectively reduced breast engorgement, breast size, and anxiety.

**What are the implications of the main findings?**

- The findings suggest that this program may help reduce fear and enhance confidence in breast care among early postpartum women.
- The implementation of this program is expected to play an important role in positively promoting the mental health and quality of life of early postpartum women.

**Abstract:**

**Background/Objectives**: Breast engorgement and associated pain, often resulting from milk stasis, impaired circulation, and tissue edema, may adversely affect maternal and neonatal health, as well as maternal mental health and quality of life in early postpartum women. Despite the availability of massage-based methods, the evidence supporting structured self-care programs remains limited. This study aims to develop and evaluate a natural breast self-circulation program and assess its effectiveness in improving breast engorgement, mental health, and quality of life among early postpartum women. **Methods**: The sample for this quasi-experimental study, comprising both an experimental group (n = 36) and a control group (n = 36), consisted of pregnant women at or before 37 weeks of gestation who intended to breastfeed. Breast engorgement and circumference, stress, anxiety, and EQ-5D-3L scores were measured before and after implementation of the intervention (the natural breast self-circulation program). **Results**: The program significantly reduced breast engorgement (F = 33.97, *p* < 0.001, partial η^2^ = 0.327), breast circumference (F = 105.52, *p* < 0.001, partial η^2^ = 0.601), and anxiety (F = 37.43, *p* < 0.001, partial η^2^ = 0.348) in women during the early postpartum period. **Conclusions**: These findings demonstrate that an early postpartum natural breast self-care program can alleviate breast engorgement and maternal anxiety. They provide a rationale for implementing self-managed breast care. Active implementation of this program may help alleviate physical and emotional difficulties and enhance confidence in breast care among pregnant women.

## 1. Introduction

Pregnancy and childbirth induce profound changes in a woman’s breast tissue, rendering breast care especially important [1]. During the postpartum period, mothers frequently experience breast engorgement because of increased milk production [2]. Without adequate breast care, women may develop discomfort, pain, blocked ducts, and mastitis, often leading to the premature cessation of breastfeeding [3,4]. Such complications can negatively affect the health of both the mother and her infant [5,6].

Additionally, specific conditions and stressors associated with pregnancy and childbirth may increase women’s vulnerability to maternal mental health problems by elevating stress and anxiety levels [7]. Physical symptoms, including breast engorgement and pain during the early postpartum period, may further worsen a mother’s already vulnerable mental health status and adversely affect her quality of life (QoL) [8,9]. These co-occurring conditions—breast engorgement, anxiety, and reduced quality of life—are not merely concurrent but mutually reinforcing, creating a compounding health burden for postpartum women. This pattern is consistent with syndemic theory, which posits that two or more health problems interact synergistically, resulting in a disease burden that exceeds what each condition would produce independently [10].

Various interventions have been employed to promote breast health, including pharmacological treatments such as ibuprofen and serrapeptase, hot and cold compresses, cabbage leaf application, acupuncture, ultrasound treatment, and massage [4,11]. However, pharmacological treatments may lead to interruption or cessation of breastfeeding because of concerns regarding potential drug-related exposure to breastfed infants [12], whereas acupuncture may provoke fear or a sense of burden in pregnant women before childbirth because of its invasive nature [13]. Consequently, non-pharmacological interventions, such as warm or cold compresses, cabbage leaves, and massage, have been widely adopted because of their relative safety and demonstrated positive effects in alleviating breast engorgement [4,14,15].

Within the context of interventions to optimize breast health, studies have described several structured breast massage programs—such as Oketani, Gua-Sha, and SMC [16,17,18,19]. These techniques typically incorporate gentle compression, rotational movements, and targeted pressure to soften breast tissue, enhance ductal stimulation, and facilitate efficient milk ejection [16]. Such practices have been linked to improved breast tissue elasticity, reduced engorgement and pain, increased blood and lymphatic flow, and enhanced milk production [17,19,20].

Despite these benefits, many massage techniques lack detailed explanations regarding their specific implementation methods or warrant a high level of manual skill, requiring extensive and specialized training. Consequently, the outcomes may vary according to the practitioner’s technical expertise [18,20,21]. Moreover, aggressive breast massage involving excessive compression has been associated with severe mastitis, which causes greater pain [22,23].

In this regard, self-breast massage may be limited by the difficulty of applying appropriate pressure and technique consistently without professional guidance. Although these concerns may support the use of professionally administered breast massage, they may simultaneously restrict self-breast massage. However, self-breast massage offers numerous advantages, including flexibility in time and setting, cost-effectiveness, and the promotion of self-efficacy through continuous self-monitoring and management; therefore, it should be more strongly recommended [24,25]. To this end, a standardized self-breast massage education program is required that is both readily replicable and universally accessible and incorporates straightforward procedures to facilitate consistent practice. Accordingly, this study develops a natural breast self-circulation program based on the principles of circulation and breathing as fundamental elements of postpartum breast care and evaluates its effect on breast engorgement, maternal mental health, and QoL in early postpartum women.

### Hypotheses

The hypotheses of this study are as follows:

**Hypothesis** **1.** *Participants in the experimental group who have completed the natural breast self-circulation program will have lower postnatal breast discomfort (breast engorgement) scores than the control group that did not receive the intervention.*

**Hypothesis** **2.** *Participants in the experimental group will show a greater reduction in breast size before and after postpartum massage than the control group.*

**Hypothesis** **3.** *Participants in the experimental group will have lower stress scores than the control group.*

**Hypothesis** **4.** *Participants in the experimental group will have lower anxiety scores than the control group.*

**Hypothesis** **5.** *Participants in the experimental group will have higher QoL scores than the control group.*

## 2. Materials and Methods

### 2.1. Study Design

This quasi-experimental study evaluated the effectiveness of a natural breast self-circulation program on postpartum breast engorgement, mental health, and QoL in pregnant women. This study adhered to the relevant EQUATOR guidelines, specifically the Transparent Reporting of Evaluations with Nonrandomized Designs (TREND) statement [26].

### 2.2. Population and Sample

The participants were primiparous women at or below 37 weeks of gestation who had not previously received breast-care education, intended to breastfeed postpartum, and voluntarily consented to participate. Individuals with communication difficulties that could have compromised educational effectiveness, or those unable or unwilling to complete the study assessments, were excluded. The participants were recruited from Dongtan Hospital located in Gyeonggi Province, South Korea, through research recruitment notices posted on the bulletin board in the obstetrics outpatient clinic, in the area next to the nurse station, and on the bulletin boards of the obstetrics ward on each floor. Participants recruited in the outpatient clinic were assigned by the researcher to either the experimental group or the control group. To reduce threats to internal validity due to the potential diffusion of the intervention, participants were admitted, excluding those in single-occupancy rooms, to obstetric wards located on different floors according to group assignment through the hospital’s routine admission procedure managed by the administrative office. Specifically, Wards A and B were assigned to the experimental group, whereas Wards C and D were assigned to the control group. Room assignment within the designated wards was based on bed availability and followed the hospital’s routine admission procedure. Group allocation was blinded to the participants. During the consent and pre-intervention education process, participants were instructed not to disclose the intervention details to others.

**Figure 1:**
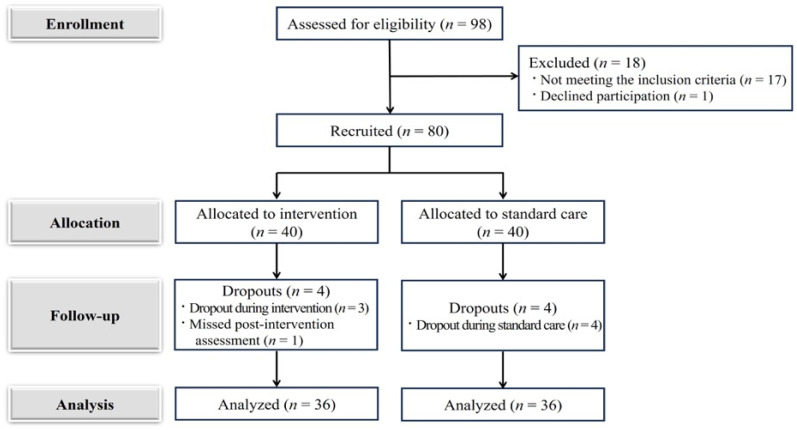
The flowchart of this study.

The required sample size was determined using G*Power version 3.1.9.7 (Heinrich-Heine-Universität Düsseldorf, http://www.gpower.hhu.de/, accessed on 23 September 2023). Based on repeated-measures analysis of variance (ANOVA) with two groups, two measurement points, a medium effect size (f = 0.25), a significance level of 0.05, and a statistical power of 0.95, the minimum required sample size was 27 participants per group. Considering that prior research has reported higher attrition rates among pregnant women compared with the typical 10–15% observed in the general population, an estimated attrition rate of 45% was applied to maintain adequate sample retention [27]. Accordingly, 80 participants were enrolled, and 40 participants were allocated to each group. During the postpartum intervention period, three participants in the experimental group and four in the control group withdrew from the study. Additionally, one participant in the experimental group failed to complete the postintervention survey. Consequently, the final analysis included 72 participants, with 36 participants in each group (Figure 1).

### 2.3. Research Setting

This study was conducted at the Department of Obstetrics and Gynecology at Dongtan Jeil Women’s Hospital in Hwaseong City, Gyeonggi Province, South Korea. Dongtan Jeil Women’s Hospital is a specialized women’s and children’s hospital with 273 inpatient beds. The hospital operates seven medical departments, including obstetrics and gynecology, and specialized centers, such as a high-risk delivery center, a high-risk pregnancy clinic, a twin pregnancy clinic, and a personalized maternal health promotion center. With more than 5000 deliveries annually, the hospital ranks among the highest-volume birth centers in South Korea.

### 2.4. Measurements

#### 2.4.1. Breast Discomfort (Breast Engorgement Scale)

Breast discomfort (engorgement) was evaluated using the 6-point Breast Engorgement Scale developed by Hill and Humenick [28]. This single-item tool assesses breast firmness and tenderness on a scale ranging from 1 (soft, no change) to 6 (very firm, severe tenderness). Because of its single-item nature, internal consistency reliability measures such as Cronbach’s α are not applicable. However, Hill and Humenick [28] used repeated measurements over a 14-day postpartum period and demonstrated a physiological pattern characterized by an initial increase in breast engorgement after delivery followed by a gradual decline, thereby supporting its predictive validity and clinical utility. In the present study, a trained researcher assessed breast engorgement scores at each measurement point using the same method.

#### 2.4.2. Breast Size

Breast size was measured using a measuring tape to evaluate breast engorgement. Measurements were obtained by marking a point 2.5 cm above the nipple at both ends of the breast base, with the longest distance recorded. Assessments were conducted at three time points: pre-delivery, post-delivery before massage, and post-delivery after massage. During the preliminary survey, both breasts are measured, and the size of the larger breast is recorded. To ensure consistency, the same breast and measurement directions were used at all three time points by the same researcher.

#### 2.4.3. Stress

Stress was assessed using the Pregnant Women’s Stress Scale developed by Ahn [29]. The tool comprises 26 items across three domains: fetal-related stress (9 items), self-related stress (11 items), and spouse-related stress (6 items). Responses are rated on a 5-point Likert scale (1–5), yielding total scores ranging from 26 to 130, with higher scores indicating greater stress levels. At the time of development, the scale demonstrated acceptable reliability (Cronbach’s α = 0.82), while the reliability in the present study was high (Cronbach’s α = 0.91).

#### 2.4.4. Anxiety

Anxiety was measured using the State–Trait Anxiety Inventory (STAI), originally developed by Spielberger [30] and later translated and culturally adapted for the Korean population by Kim and Shin [31]. The state anxiety subscale consists of 20 items rated on a 4-point Likert scale, yielding total scores ranging from 20 to 80. Higher scores reflect greater levels of anxiety. The reliability of the tool at the time of its development was Cronbach’s α = 0.87, and the reliability in the present study was Cronbach’s α = 0.93.

#### 2.4.5. Quality of Life

QoL was assessed using the EQ-5D-3L, a health-related quality of life (HRQoL) instrument developed by the EuroQol Group [32]. The measure encompasses five independent dimensions: mobility, self-care, usual activities, pain/discomfort, and anxiety/depression. Each dimension is rated on three levels: “no problems” (1 point), “some problems” (2 points), and “extreme problems” (3 points). Rather than calculation of a simple sum, the score is calculated using country-specific value sets to derive an index score. In this study, the index was calculated using the Korean value set that reflects population preferences [33].

### 2.5. The Natural Breast Self-Circulation Program

The natural breast self-circulation program is an educational intervention designed to enable pregnant women to perform self-breast care through self-administered breast massage therapy. The program education was delivered at three time points: approximately two to three weeks prior to the expected delivery date (prepartum), immediately after childbirth (postpartum), and again at two weeks postpartum. The program is grounded in the traditional East Asian medicine concept that physiological and environmental changes during pregnancy [1,2,21], combined with stress associated with childbirth and the early postpartum period, may predispose women to pathogenic cold (han, 寒), a traditional medicine pattern associated with contraction and impaired flow. Within this framework, such changes are thought to increase breast tension, restrict circulation, and exacerbate milk stasis and engorgement [34]. Accordingly, the program seeks to preemptively resolve this cold state by creating a circulation-conducive environment that enhances milk flow, facilitates milk ejection, and ultimately reduces breast engorgement [35].

**Figure 2:**
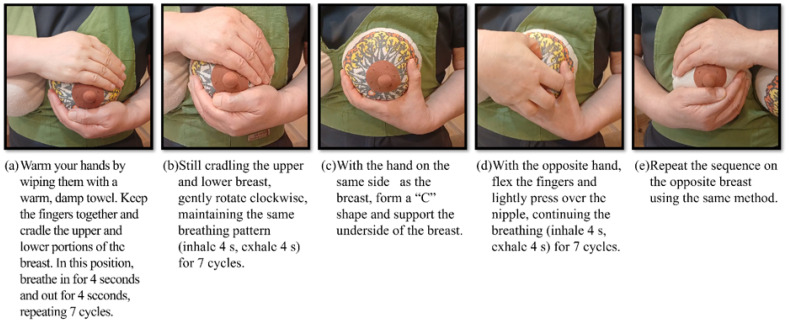
Detailed Steps of the Natural Breast Self-Circulation Program.

To this end, slow diaphragmatic breathing is incorporated throughout all steps to stimulate the parasympathetic nervous system, reduce stress and anxiety, promote thoracic relaxation, and enhance peripheral blood and lymphatic flow [36]. The procedural framework consists of four steps: preparation, circulation, ejection, and stabilization, and comprises two phases: (1) prepartum preparation and (2) postpartum circulation activation. Participants first received an explanation of the program’s rationale, then observed a demonstration using a model. Finally, guided coaching was provided so that each participant could apply the method independently. Details of the natural breast self-circulation method are presented in Figure 2.

#### 2.5.1. Prepartum Preparation Phase

The program was conducted twice daily (morning and afternoon) and targeted pregnant women at 37–38 weeks’ gestation. The objective was to establish the foundation for breast circulation and facilitate effective circulation after childbirth.

#### 2.5.2. Postpartum Circulation Activation Phase

The self-breast circulation phase was implemented during the period of robust milk output, with one session before and after each feeding. If we assume an average of eight feedings per day, the daily target was 16 sessions. To confirm completion of these phase sessions, the researcher visited postpartum care centers in person for participants staying there and confirmed completion for those receiving postpartum care at home via mobile phone calls or text messages.

### 2.6. Standard Care

Participants in the control group received only standard care education that is routinely provided to all postpartum women. This education included general breastfeeding guidance (accurate latch technique, frequency, and duration of feeding), the importance of frequent milk removal, potential risks associated with excessive pumping, principles of thermal therapy (warm application before feeding and cold application afterward), fundamental breast skin care and hygiene, and instructions regarding follow-up and warning signs of breast complications. Education was provided on the day of delivery and reinforced at hospital discharge.

### 2.7. Data Collection Methods and Procedures

The data collection period extended from 1 December 2023, to 30 September 2024. The pre-intervention survey was conducted in separate wards for both the experimental and control groups. The researcher personally distributed the presurvey questionnaire, which included items assessing demographic characteristics, breast discomfort, stress, anxiety, and QoL. Participants were instructed to complete the questionnaire in a private and secure setting to ensure anonymity. Procedures for breast size measurement were explained in advance, and measurements were taken directly by the researcher using a measuring tape.

The natural self-breast circulation program was implemented at 37 and 38 weeks of gestation, on the day of delivery, and again at two weeks postpartum in the hospital’s program and education rooms. Although the program was originally developed for groups of up to ten participants, sessions were conducted in smaller groups of one to three participants, with each session lasting approximately 20 min.

The post-intervention survey was administered at two weeks postpartum, consistent with previous research [25], to facilitate data collection after the participants had returned home from postpartum care centers. The researcher personally distributed the post-survey questionnaire, which included only the dependent variables and excluded demographic items. The instructions and precautions for completing the questionnaire were explained verbally, and participants completed the questionnaire independently. Breast size measurements during the post-intervention survey were taken both before and after the self-breast massage to permit comparison. Completion of the pre- and post-surveys and breast measurements took approximately 15–20 min respectively. After completion, the researcher checked for incomplete responses or missing items, requested additional input if necessary, and collected the questionnaires on-site once they were fully completed.

### 2.8. Data Analysis Methods

All data were analyzed using IBM SPSS Statistics (version 31.0; IBM Corp., Armonk, NY, USA). Descriptive statistics, including frequencies, percentages, means, and standard deviations, were used to summarize the participants’ general characteristics. The normality of the dependent variables was assessed using the Shapiro–Wilk test. Homogeneity tests between groups with respect to general characteristics and dependent variables were conducted using the chi-square test, independent *t*-test, and Mann–Whitney U test. Program effectiveness was evaluated using repeated-measures ANOVA, and the effect size of the intervention program was calculated using partial η^2^ [37]. A partial η^2^ value of ≤0.01 was interpreted as a small effect size, 0.06 as medium, and ≥0.14 as large.

### 2.9. Ethical Consideration

Participants who voluntarily wished to participate in the study, as indicated in the ward notice, were provided with a written informed consent form, and a signed copy was provided to each participant. All collected data were used exclusively for research purposes, and strict measures were implemented to maintain anonymity and confidentiality. Participants were informed that refusal to participate would result in no disadvantage and that withdrawal was permitted at any time. If counseling related to the study was requested, both participants and their guardians were offered appropriate consultation. After the study concluded, participants in the control group were informed that they could receive the same intervention program upon request. Additionally, all participants in the experimental and control groups who completed the questionnaire and participated in the program received a small gift as a token of appreciation.

## 3. Results

### 3.1. Homogeneity Test of Participants’ General Characteristics and Baseline Dependent Variables

The homogeneity test of the general characteristics between the experimental and control groups revealed no significant differences in age, marital status, educational attainment, occupation, income level, history of miscarriage, maternal health status, fetal health status, medication use, postpartum caregiving plans, and marital satisfaction, indicating that the two groups were homogeneous (Table 1). The Shapiro–Wilk test was conducted to evaluate the normality of the dependent variables. Results demonstrated that breast size, stress, and anxiety were normally distributed (*p* = 0.144–0.972), whereas breast discomfort and QoL did not meet the normality assumptions. However, breast discomfort scores demonstrated Fisher’s skewness values within ±1.96 and were therefore considered approximately normally distributed. Accordingly, independent *t*-tests were performed for normally distributed variables (breast discomfort, breast size, stress, and anxiety), while the Mann–Whitney U test was used to analyze QoL. No statistically significant between-group differences were identified, confirming homogeneity (Table 1). Regarding postpartum obstetric characteristics, the distribution of delivery mode was similar between the two groups. In the experimental group, 22 women underwent cesarean section and 14 had vaginal delivery, whereas in the control group, 18 underwent cesarean section and 18 had vaginal delivery. No participant in either group discontinued breastfeeding during the study period.

### 3.2. Effectiveness of the Natural Breast Self-Circulation Program

Analysis of Hypothesis 1 demonstrated a statistically significant between-group difference in breast discomfort (engorgement) scores (F = 5.75, *p* = 0.019, partial η^2^ = 0.076), indicating a medium effect size. Although the main effect of time was not statistically significant (F = 0.06, *p* = 0.801, partial η^2^ = 0.010), the interaction effect between group and time showed statistical significance (F = 33.97, *p* < 0.001, partial η^2^ = 0.327), thereby supporting Hypothesis 1. The large effect size observed for the interaction effect indicates that the natural breast self-circulation program significantly reduced breast discomfort compared with the control group (Table 2).

Testing of Hypothesis 2 revealed that the difference in breast size scores before and after postpartum self-circulation program was not statistically significant between groups (F = 2.975, *p* = 0.089, partial η^2^ = 0.041). However, significant differences over time (F = 103.58, *p* < 0.001, partial η^2^ = 0.597) and a significant interaction effect between group and time (F = 105.52, *p* < 0.001, partial η^2^ = 0.601) were observed, indicating that the natural breast self-circulation program positively influenced breast size changes. Therefore, Hypothesis 2 was supported. Additionally, when prepartum and postpartum measurements obtained before massage were compared, breast size increased significantly because of childbirth (F = 252.86, *p* < 0.001, partial η^2^ = 0.783), with no significant between-group differences (F = 0.62, *p* = 0.434, partial η^2^ = 0.009) and no significant interaction effect (F = 0.16, *p* = 0.690, partial η^2^ = 0.002). In a comparison of prepartum and postpartum measurements obtained after massage, significant differences were observed between groups (F = 5.08, *p* = 0.027, partial η^2^ = 0.068), over time (F = 112.17, *p* < 0.001, partial η^2^ = 0.616), and in the group-by-time interaction effect (F = 68.85, *p* < 0.001, partial η^2^ = 0.496). These findings indicate that the experimental group experienced a greater post-massage reduction in breast size than the control group (Table 3).

The analysis of Hypothesis 3 revealed that the stress scores did not differ significantly between groups (F = 1.59, *p* = 0.212, partial η^2^ = 0.022), across time points (F = 1.55, *p* = 0.217, partial η^2^ = 0.022), or in the group × time interaction (F = 1.35, *p* = 0.249, partial η^2^ = 0.019). In the experimental group, the mean stress score decreased from 38.17 ± 9.98 at pretest to 30.78 ± 6.51 at posttest, whereas in the control group, the mean score changed from 37.14 ± 9.37 to 39.42 ± 8.03. However, as the breast self-circulation program did not show a statistically significant effect on stress (Table 2), Hypothesis 3 was not supported.

The analysis of Hypothesis 4 revealed that anxiety scores showed statistically significant effects between groups (F = 4.18, *p* = 0.045, partial η^2^ = 0.056), across time points (F = 10.46, *p* = 0.002, partial η^2^ = 0.130), and for the group × time interaction (F = 37.43, *p* < 0.001, partial η^2^ = 0.348), thereby supporting Hypothesis 4. Notably, the interaction effect was large, suggesting that the natural breast self-circulation program had a strong impact on anxiety scores (Table 2).

The analysis of Hypothesis 5 indicated that the QoL scores did not satisfy normality assumptions; therefore, nonparametric tests were conducted. The Wilcoxon signed-rank test examining pre–post changes within each group revealed no statistically significant differences in either the experimental group (Z = −0.10, *p* = 0.917, r = 0.017) or the control group (Z = −1.48, *p* = 0.138, r = 0.247). Moreover, the Mann–Whitney U test showed no significant between-group differences in QoL score changes (U = 527.00, Z = −1.40, *p* = 0.163, r = 0.165). Accordingly, Hypothesis 5 was rejected (Table 2).

## 4. Discussion

This study aimed to develop a prenatal and postnatal natural breast self-circulation program for breast care and evaluated its effects on breast engorgement, mental health, and QoL among pregnant women. The results are as follows.

First, education in the natural breast self-circulation program significantly reduced breast engorgement during the early postpartum period. This finding was generally consistent with the results reported by Park [25] on self-breast massage and by Chiu et al. [19] on Gua-Sha breast massage, both of which demonstrably reduced breast engorgement. However, variations in massage protocols and measurement scales for breast engorgement across studies have limited direct comparisons. Since breast engorgement is usually caused by milk stasis, impaired blood and lymphatic circulation, and tissue edema, the natural breast self-circulation program developed in this study was designed to address these underlying mechanisms. Specifically, manual massage techniques were applied to stimulate lymphatic flow and blood circulation [5], internal coldness was removed to restore the flow of qi and blood, thereby facilitating the opening of milk ducts [35], and breathing techniques were used to regulate the autonomic nervous system [36]. Collectively, these factors may have contributed to the observed reduction in breast engorgement. However, no significant difference was found between the two groups at identical time points (F = 0.06, *p* = 0.801, partial η^2^ = 0.010), likely reflecting the increased breast engorgement observed in the early postpartum period. Providing clear education regarding this physiological change during the natural breast self-circulation program may enhance postpartum breast self-care.

Second, the program produced reduced breast size before and after massage in the early postpartum period, objectively demonstrating its effectiveness in reducing breast engorgement. This finding aligned with the results of Cho and Ahn [38], which reported that a breastfeeding promotion program incorporating basic breast massage significantly reduced breast size. When participants confirmed a reduction in breast size before and after the program, their trust in the program appeared to increase, as did their willingness to continue participation. This observation is consistent with the findings of Seewoodharry et al. [39], which demonstrated that objective feedback enhances performance. Accordingly, incorporating pre- and post-intervention breast size measurements into early postpartum breast self-circulation education may strengthen participants’ confidence and promote adherence to the intervention.

Third, the program did not significantly reduce postpartum stress between the experimental and control groups. In contrast to Ramezani et al. [40], who reported significant stress reduction following breast massage in primiparous women, the present study observed decreased stress within the experimental group and relatively stable stress levels within the control group, suggesting a potential benefit of natural breast self-circulation therapy. The early postpartum period is characterized by concurrent physical, emotional, and environmental stressors, each of which may attenuate the program’s effects. The early postpartum period is a time when physical, emotional, and environmental stresses occur simultaneously. Considering that postpartum stress is a multidimensional variable influenced not only by breast symptoms but also by various relational factors such as social support, marital satisfaction, and mother–infant attachment, the lack of a significant difference in stress reduction between the two groups in this study may be interpreted as the intervention effect being partially diluted within the complex stress structure of the early postpartum period [41]. Therefore, future programs should consider expanding this program into a postpartum stress intervention program that goes beyond a symptom-relief approach to also incorporate social support and relational contexts.

Fourth, the program significantly reduced anxiety in women during the early postpartum period. This finding, which was consistent with research documenting the anxiolytic effects of massage during pregnancy and childbirth, suggests that breast-focused interventions may be particularly effective [42,43,44]. Kilci Erciyas and Kavlak [44] similarly reported reduced anxiety among mothers of preterm infants following combined breast and back massage; however, the present study extends prior findings by exclusively exploring the effects of an intervention focused solely on the breast area. The natural breast self-circulation program is presumed to promote oxytocin secretion and activation of the parasympathetic nervous system via tactile stimulation of the breast and nipple areas combined with breathing exercises [1,36]. As breast engorgement and tenderness diminished, anticipatory anxiety or negative emotions linked to physical discomfort likely decreased, thereby contributing to emotional stabilization [3,4,28]. Additionally, the self-administered nature of the program may have strengthened the participants’ perceived control and self-efficacy, thereby contributing to the reduced anxiety. Through the combined influence of these multifaceted factors, the present intervention was found to be particularly useful for pregnant and postpartum women who were especially vulnerable to heightened anxiety during pregnancy and the early postpartum period.

Finally, there was no statistically significant between-group difference in QoL among women during the early postpartum period. Studies indicate that physical injuries sustained during childbirth (including those related to the mode of delivery and physiological changes), common breastfeeding-related complications (such as breast engorgement or pain), sleep deprivation, fatigue associated with newborn care, and emotional variability can all critically compromise women’s QoL in the early postpartum period, often resulting in a decline in overall QoL [45,46]. Within this context, the cumulative physical, emotional, and situational challenges women face in the early postpartum period may attenuate the potential effects of the intervention on their QoL. Kohler et al. reported that HRQoL utility scores are typically low within the first seven days postpartum and tend to recover naturally by approximately three weeks after delivery [47]. Therefore, the early postpartum period represents a time during which mothers experience decreased HRQoL, with rapid changes over time. Additionally, it is important to note that the EQ-5D-3L HRQoL instrument employed in this study generates scores in which values closer to 1 represent higher QoL [32]. In the experimental group, HRQoL scores remained stable (0.94 ± 0.07 before childbirth vs. 0.94 ± 0.10 after childbirth), demonstrating minimal change between the prenatal and postpartum periods. In contrast, the control group exhibited a decline from 0.93 ± 0.13 before childbirth to 0.89 ± 0.17 after childbirth. This finding indicates that in the context of a general decline in QoL scores among women in the early postpartum period, the program may have contributed to maintaining QoL scores. However, postpartum QoL is a multidimensional outcome shaped not only by breast-related symptoms, but also by marital or relationship context and educational level. These factors may influence emotional and practical support, health literacy, and self-management capacity, which in turn may have limited the extent to which the intervention effect was reflected in between-group QoL differences [48]. Therefore, future studies should take into account demographic and contextual factors such as marital status and education level and assess the long-term effects of breast care interventions on quality of life.

The present study has several limitations that should be considered. First, because data were collected from a single institution in South Korea and the sample size was limited, the generalizability of the findings may be limited. Second, a quasi-experimental design, rather than a randomized controlled trial (RCT), limits the ability to fully control for potential confounding variables in the between-group comparisons. Third, although baseline comorbidities and current medication use were assessed, postpartum treatments and pre-existing mental health disorders were not specifically assessed. Therefore, their effects on the outcomes cannot be excluded. Fourth, the follow-up period was limited to approximately two weeks postpartum, making it difficult to determine the long-term effects of the program.

## 5. Conclusions

This study developed and evaluated a prenatal and postnatal breast care program, the natural breast self-circulation program, and examined its effects on postpartum breast engorgement, mental health (stress and anxiety), and QoL among pregnant women. The findings confirmed that the program was effective in reducing breast engorgement and anxiety in the early postpartum period (partial η^2^ = 0.327–0.601). By enabling mothers to independently manage their breast health, the program may strengthen a critical foundation for maternal and infant well-being during pregnancy and childbirth while simultaneously fostering awareness of the importance of self-care. Accordingly, along with the development of a system to monitor continuous breast care from the prenatal to postpartum periods, the integration of this program may help reduce fear, enhance confidence in breast care, and lower additional breast care-related costs, thereby suggesting its potential as a cost-effective intervention for pregnant and postpartum women.

Future studies should replicate the natural breast self-circulation program across diverse institutions and countries, preferably using RCT designs. Furthermore, extending the observation period throughout the postpartum stage would allow verification of the program’s long-term effects while incorporating modifications such as increasing the number of educational sessions within the program and expanding the number of measurement time points. In addition, the relevancy of sociodemographic and clinical factors interacting with program effectiveness should be considered.

## Figures and Tables

**Table 1:** Homogeneity test of participants’ general characteristics and baseline dependent variables.

Characteristics and Baseline Dependent Variables	EG (n = 36) M ± SD or N (%)	CG (n = 36) M ± SD or N (%)	χ^2^ or *t* *	*p*-Value
Age (yr)	33.06 ± 3.70	33.81 ± 3.70	12.93	0.741
Marital status			1.01	0.314
Married	35 (97.2)	36 (100.0)		
Cohabiting	1 (2.8)	-		
Educational level			0.86	0.652
High school graduate	2 (5.6)	2 (5.6)		
College graduate	29 (80.5)	26 (72.2)		
Postgraduate degree	5 (13.9)	8 (22.2)		
Occupation			2.67	0.102
Yes	30 (83.3)	24 (66.7)		
No	6 (16.7)	12 (33.3)		
Income level (Monthly)			7.01	0.135
Less than 4 million KRW (≈USD 3000)	5 (13.9)	6 (16.7)		
4~6 million KRW (≈USD 3000~4500)	17 (47.2)	12 (33.3)		
More than 6 million KRW (≈USD 4500)	14 (38.9)	18 (50.0)		
History of miscarriage			1.29	0.257
Yes	10 (27.8)	6 (16.7)		
No	26 (72.2)	30 (83.3)		
Health status			5.27	0.261
Poor health	3 (8.3)	2 (5.6)		
Fair health	13 (36.1)	7 (19.4)		
Good health	20 (55.6)	27 (75.0)		
Fetal health status			1.01	0.314
Good	35 (97.2)	36 (100.0)		
Abnormal	1 (2.8)	-		
Use of pharmacological treatment			0.08	0.770
Yes	7 (19.4)	8 (22.2)		
No	29 (80.6)	28 (77.8)		
Postpartum caregiving plan (Within 2 weeks)			2.22	0.529
Family	2 (5.6)	3 (8.3)		
Postpartum care center	32 (88.8)	33 (91.7)		
Children helper	2 (5.6)	-		
Marital satisfaction	9.50 ± 0.91	9.31 ± 1.09	4.40	0.355
Breast size	8.93 ± 1.06	8.74 ± 1.06	0.72	0.473
Breast discomfort	2.53 ± 1.00	2.31 ± 0.82	1.03	0.306
Stress	58.94 ± 14.43	60.92 ± 13.91	−0.59	0.557
Anxiety	38.17 ± 9.98	37.14 ± 9.37	0.45	0.654
Quality of life	0.94 ± 0.07	0.92 ± 0.13	−0.03 *	0.976

EG, experimental group; CG, control group. * Mann–Whitney U test.

**Table 2:** Change in breast discomfort, stress, anxiety, and quality of life between two groups.

Variables	Group	Pre-Test M ± SD	Post-Test M ± SD	Source	F *	*p*-Value (Partial η^2^)
Breast discomfort	EG (n = 36)	2.53 ± 1.00	1.92 ± 0.60	G	5.75	0.019 (0.076)
	CG (n = 36)	2.31 ± 0.82	2.97 ± 1.00	T	0.06	0.801 (0.010)
				G × T	33.97	<0.001 (0.327)
Stress	EG (n = 36)	58.94 ± 14.43	54.92 ± 14.88	G	1.59	0.212 (0.022)
	CG (n = 36)	60.92 ± 13.91	60.78 ± 16.60	T	1.55	0.217 (0.022)
				G × T	1.35	0.249 (0.019)
Anxiety	EG (n = 36)	38.17 ± 9.98	30.78 ± 6.51	G	4.18	0.045 (0.056)
	CG (n = 36)	37.14 ± 9.37	39.42 ± 8.03	T	10.46	0.002 (0.130)
				G × T	37.43	<0.001 (0.348)
Quality of life	EG (n = 36)	0.94 ± 0.07	0.94 ± 0.10	In-G (Wilcoxon)	−0.10	0.917 (0.017)
	CG (n = 36)	0.93 ± 0.13	0.89 ± 0.17		−1.48	0.138 (0.247)
				U(Z)	527.0 * (−1.40)	0.163 (0.165) ^†^

EG, experimental group; CG, control group; G, group; T, time; G × T, interaction term between group and time; In-G, within-group; U(Z), Mann–Whitney U score (Z score). * Mann–Whitney U test. ^†^ Effect size r (rank-biserial correlation).

**Table 3:** Comparison of breast size score between two groups.

Group	Prepartum M ± SD		Postpartum (Before Self-Circulation Program) M ± SD	Postpartum (After Self-Circulation Program) M ± SD
Experimental Group (n = 36)	8.93 ± 1.06		10.96 ± 1.50	9.16 ± 1.28
Control Group (n = 36)	8.74 ± 1.06		10.68 ± 1.67	10.69 ± 1.74
**Comparison**	**Source**	**F**	***p*** **-Value**	**Partial η^2^**
Postpartum (Before vs. After self-circulation program)	G	2.975	0.089	0.041
	T	103.58	<0.001	0.597
	G × T	105.52	<0.001	0.601
Prepartum vs. Postpartum (Before self-circulation program)	G	0.62	0.434	0.009
	T	252.86	<0.001	0.783
	G × T	0.16	0.690	0.002
Prepartum vs. Postpartum (After self- circulation program)	G	5.08	0.027	0.068
	T	112.17	<0.001	0.616
	G × T	68.85	<0.001	0.496

G, group; T, time; G × T, interaction term between group and time.

## Data Availability

The data presented in this study are available on request from the corresponding author due to legal or ethical reasons.

## Abbreviations

The following abbreviations are used in this manuscript:

QoL	Quality of life
TREND	Transparent reporting of evaluations with nonrandomized designs
ANOVA	Analysis of variance
STAI	State–Trait Anxiety Inventory
HRQoL	Health-related quality of life
RCT	Randomized controlled trial
